# Right-Sided Colonic Diverticulitis Mimicking Acute Appendicitis in a Pediatric Patient: A Case Report

**DOI:** 10.7759/cureus.77515

**Published:** 2025-01-15

**Authors:** Takayuki Fujii, Aya Tanaka, Hiroto Katami, Ryuichi Shimono

**Affiliations:** 1 Pediatric Surgery, Kagawa University, Takamatsu, JPN

**Keywords:** acending colonic diverticulitis, acute abdomen, diagnosis, right lower quadrant pain, ultrasonography

## Abstract

Colonic diverticulitis is rare in pediatric populations. In cases of right-sided colonic diverticulitis, the symptoms can mimic those of acute appendicitis, leading to potential misdiagnosis of acute appendicitis. We report a case of ascending colonic diverticulitis in a 15-year-old young man who presented with abdominal pain migrating from the epigastrium to the right lower quadrant, initially raising suspicion for acute appendicitis. The patient presented to a local clinic with worsening pain and was referred to our department due to right lower quadrant tenderness and an elevated white blood cell count. Contrast-enhanced computed tomography revealed a solitary diverticulum in the ascending colon, accompanied by wall thickening and pericolic fat stranding, confirming the diagnosis of diverticulitis. The patient was successfully treated with intravenous antibiotics, and his symptoms resolved without surgical intervention. This case highlights the importance of considering right-sided diverticulitis in the differential diagnosis of right lower quadrant pain, particularly in regions where it is more prevalent. Additionally, diverticulitis should be included in the differential diagnosis when evaluating patients with pain that migrates from the epigastrium to the right lower quadrant. Imaging modalities, particularly computed tomography, may play a vital role in distinguishing this condition from acute appendicitis.

## Introduction

Colonic diverticulitis is an inflammatory condition that predominantly affects the elderly in Western countries but is rare in pediatric populations [1]. Studies on colonic diverticulitis in pediatric patients are scarce, and the exact incidence remains unknown. In Western countries, it primarily affects the left side of the colon [2]. However, in East Asian populations, approximately 70% of colonic diverticulitis cases occur in the right side of the colon [3]. Right-sided diverticula are believed to develop congenitally as a result of the protrusion of all layers of a weakened intestinal wall [3]. On the other hand, left-sided diverticula are typically associated with acquired factors such as dietary habits, chronic constipation, elevated colonic pressure, defecation patterns, and inflammatory bowel disease. As a result, left-sided diverticulosis is more frequently observed in older individuals. The etiology behind these regional differences is not yet clearly understood.

In cases of right-sided colonic diverticulitis, symptoms such as right lower quadrant pain, fever, and vomiting often mimic those of acute appendicitis, leading to potential misdiagnosis of acute appendicitis [4]. We report a case of ascending colon diverticulitis in a pediatric patient who presented with migrating pain from the epigastrium to the right lower quadrant, necessitating differentiation from acute appendicitis.

## Case presentation

A generally healthy 15-year-old Japanese boy presented with a chief complaint of right lower quadrant pain. The pain initially began in the epigastric region earlier in the day and gradually migrated to the right lower abdomen. He also reported that walking exacerbated the pain. Upon examination at a local clinic, mild tenderness in the right lower quadrant and a slight elevation in white blood cell count were noted, leading to the suspicion of acute appendicitis. The patient was subsequently referred to our department for further evaluation.

On examination, the patient appeared stable, with a temperature of 37.2°C, blood pressure of 120/71 mmHg, heart rate of 72 bpm, and oxygen saturation of 99%. Abdominal examination revealed mild tenderness at McBurney's point without rebound tenderness or guarding. No palpable mass was observed. Laboratory tests revealed a white blood cell count of 11,900/µL and a C-reactive protein level of 0.2 mg/dL (Table 1).

**Table 1:** Laboratory values of the patient RBC: red blood cell, CRP: C-reactive protein, AST: aspartate aminotransferase, ALT: alanine aminotransferase, ALP: alkaline phosphatase, LDH: lactate dehydrogenase, GGT: gamma-glutamyl transferase

Laboratory parameters	On arrival	Third day	Reference ranges
White blood cells (×10³/µL)	11.9	6.2	3.9-9.8
Lymphocytes (/µL)	2374	2241	1200-5200
Neutrophil (/µL)	7786	3318	1800-8000
Hemoglobin (g/dL)	15	13	12.6-16.5
Platelets (×10^4^/µL)	17.5	16.7	17-41
RBC (×10⁶/µL)	5.1	4.4	4.3-5.6
CRP (mg/dL)	0.2	3.3	0-0.2
Urea (mg/dL)	9.2	5.8	6.8-18.8
Creatinine (mg/dL)	0.6	0.6	0.4-1
Bilirubin, total (mg/dL)	1	1.1	0.1-1.2
AST (U/L)	23	24	14-30
ALT (U/L)	21	13	9-35
ALP (U/L)	600	444	270-1200
LDH (U/L)	166	187	130-250
GGT (U/L)	18	15	9-48
Sodium (mmol/L)	141	137	135-146
Potassium (mmol/L)	4.4	4.4	3.5-4.6
Chloride (mmol/L)	104	103	96-110

Ultrasonography revealed an area resembling a fecalith; however, the appendix was not visualized.

**Figure 1:**
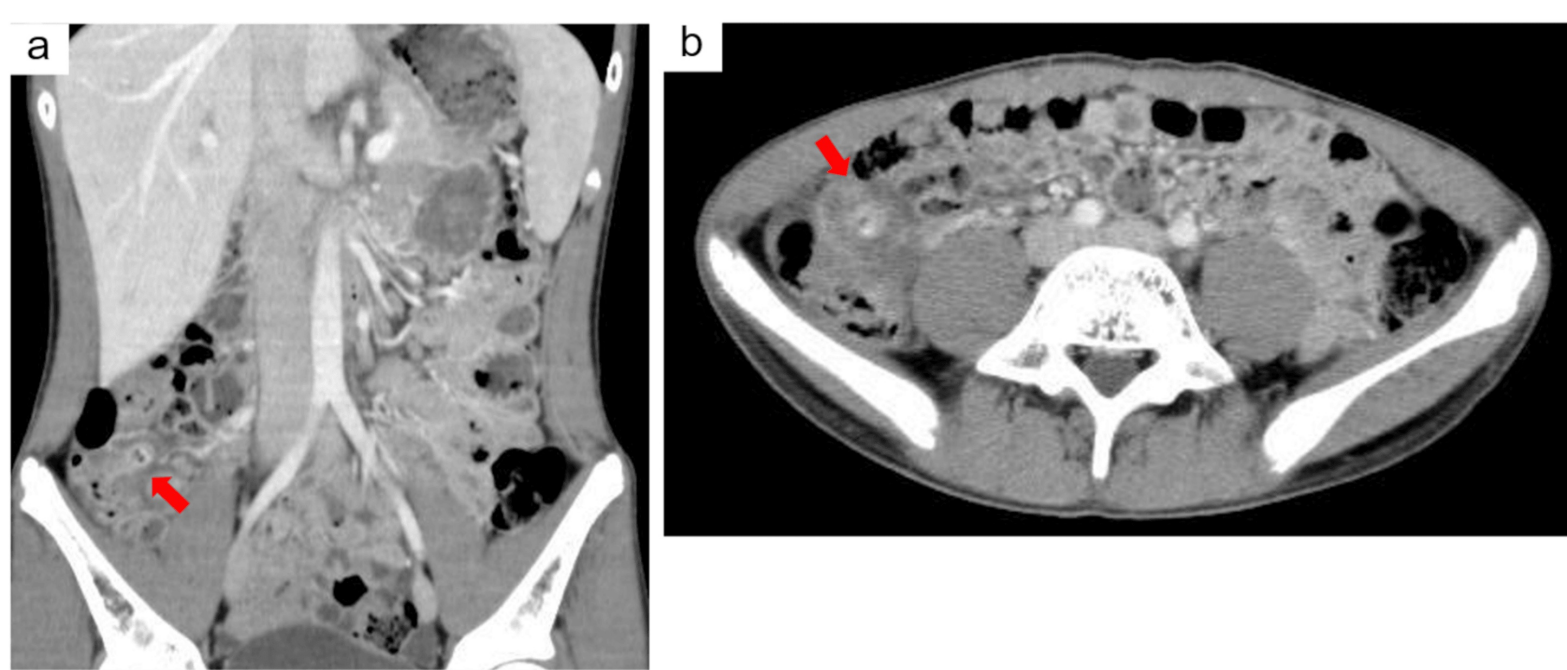
Contrast-enhanced abdominal computed tomography images (a) Coronal section: A solitary diverticulum was observed in the ascending colon, accompanied by wall thickening and increased density of the surrounding adipose tissue (arrow). (b) Axial section: A diverticulum with wall thickening and pericolic fat stranding (arrow).

Contrast-enhanced computed tomography showed no evidence of appendiceal enlargement or wall thickening. However, a single diverticulum with wall thickening and associated pericolic fat stranding was identified in the ascending colon, leading to a diagnosis of diverticulitis (Figure 1a, 1b).

The patient was admitted and placed on bowel rest. Empiric intravenous antibiotic therapy (cefmetazole) was initiated. By the third day, the patient demonstrated significant improvement, with reduced abdominal pain. Oral intake was gradually resumed. Intravenous antibiotic therapy was completed on the fifth day, and the patient was discharged on the seventh day in good condition.

## Discussion

Diverticulitis is more common in older adults; its occurrence in the pediatric population remains poorly understood. In adults, left-sided colonic diverticula are often considered acquired pseudodiverticula resulting from prolonged and repeated increases in intraluminal pressure, causing protrusion of weak areas in the intestinal wall. These diverticula are often multiple. Conversely, right-sided colonic diverticula, occasionally observed as part of acquired multiple diverticulosis, are frequently described as congenital or solitary true diverticula [5].

Abnormal colonic motility is considered a significant contributing factor in the development of diverticulitis [3]. Additionally, various other elements, including reduced physical activity, chronic constipation, obesity, smoking, and the use of nonsteroidal anti-inflammatory drugs, may interact and exacerbate the risk of acute diverticulitis [3]. However, none of these factors were applicable in the present case. Instead, the presence of a fecalith was identified, which is presumed to be the underlying cause of the diverticulitis.

The incidence of right-sided colonic diverticulitis is increasing among children and adolescents, making it an important condition to consider in the differential diagnosis of abdominal pain [4]. A multicenter study in Japan involving 1,112 participants found that right-sided colonic diverticulitis was more prevalent in individuals aged <40 years [3]. This condition frequently causes right lower quadrant pain, posing a diagnostic challenge in distinguishing it from acute appendicitis. Similarly, a Korean study of 104 children and adolescents reported that 86% presented with right lower quadrant pain, and 6% were misdiagnosed with acute appendicitis, resulting in unnecessary appendectomy [4]. To prevent unnecessary appendectomies, it is essential to establish an accurate preoperative diagnosis through imaging studies and consider the possibility of diverticulitis intraoperatively when the appendix is found to be normal.

The migration of pain from the epigastrium to the right lower quadrant observed in this case likely follows a mechanism similar to that observed in appendicitis. In acute appendicitis, patients often initially experience colicky pain in the periumbilical region that intensifies over the first 24 hours and transitions into a sharp and constant pain localized to the right iliac fossa. This progression occurs as visceral pain transitions into somatic pain [6,7]. Visceral pain arises from receptors located in the muscles and mucosa of hollow organs, as well as the mesentery and serosal surfaces, which are primarily activated by stretching or torsion. This type of pain is typically diffuse and poorly localized due to the characteristics of its afferent nerves, which are sparse, unmyelinated, and bilateral and enter the spinal cord at multiple levels. In contrast, somatoparietal pain originates from receptors in the parietal peritoneum, muscles, and skin and is triggered by inflammation or stretching. Unlike visceral pain, somatoparietal pain is sharp, well-localized, and more intense, as it is transmitted by numerous myelinated nerves that connect to specific dorsal root ganglia [7].

Ultrasonography and computed tomography have reported sensitivities of 91% and 93%, respectively [8], for diagnosing right-sided colonic diverticulitis, both of which are considered relatively high. However, ultrasonography is subject to operator dependency, affecting the reliability and validity of measurements [9]. Thus, when ultrasonography alone is inconclusive and differentiation from appendicitis is required, computed tomography is becoming essential [10].

Han et al. reported that intravenous antibiotics were administered in 68% of diverticulitis cases, whereas 24% of patients were adequately managed with oral antibiotics [4]. The recurrence rate in patients with inflammation was 7.8%; however, all patients were successfully managed with conservative treatment [4]. There are a few reports in the literature indicating that surgical intervention has been performed in pediatric patients with right-sided diverticulitis [4,10]. The severity of diverticulitis in children and adolescents is generally lower than that in adults, making conservative treatment a viable and effective option [4,11]. The lower severity of diverticulitis in children and adolescents is likely related to their overall lower risk of complications compared to adults. This difference may be attributed to anatomical and physiological factors, as well as a less intense inflammatory response observed in younger individuals [4].

## Conclusions

Right-sided colonic diverticulitis, although rare in pediatric populations, must be considered when evaluating right lower quadrant pain with epigastric migration. Computed tomography may play a vital role in distinguishing this condition from acute appendicitis.
